# Bioactive compounds in Chinese herbal medicine: anti-inflammatory mechanisms targeting neurological disorders

**DOI:** 10.3389/fnut.2025.1646438

**Published:** 2025-10-01

**Authors:** Liangxue Zhang, Jiaxin Yang, Yuhua Yang, Min Yang, Juan Yang, Changyin Yu, Haiqing Zhang, Jinmei Tuo, Zucai Xu

**Affiliations:** ^1^Department of Neurology, Affiliated Hospital of Zunyi Medical University, Zunyi, China; ^2^Guizhou Provincial Key Laboratory of Brain Function and Prevention and Treatment of Guizhou Province, Zunyi, China; ^3^Department of Nursing, Affiliated Hospital of Zunyi Medical University, Zunyi, China

**Keywords:** herbal medicine, neuroinflammation, molecular mechanisms, neurological disorders, multi-target therapy, gut-brain axis

## Abstract

Nearly 16% of the world’s population is affected by neurological disorders, including neurodegenerative and neuroimmune diseases caused by acute or chronic inflammation. Inflammatory processes in the central nervous system can exacerbate these diseases by causing neuronal damage and apoptosis. Traditional Chinese medicines have become an important area of research in anti-neuroinflammation and neuroprotection owing to their multi-target effects and favorable safety profiles. In this paper, we review the molecular mechanisms by which bioactive compounds of herbal origin inhibit neuroinflammation and improve disease progression through the modulation of inflammatory factors (including TLR4/MyD88/NF-κB, NLRP3 inflammasomes, and Janus kinase-STAT signaling), epigenetic modifications, cell-type-specific modulation (microglia M1/M2 polarization and astrocyte A1/A2 transformation), and gut-brain axis interactions. These bioactive compounds are mainly classified into those with well-defined chemical structures (such as baicalein, baicalin, berberine, and ginsenoside Rg1), plant extracts (such as tonifying Yang Huiwu Tang, Tongxinluo capsule, Shu Xuning injection, and Xingxiong injection), and preparations based on special mechanisms of action or technical means (such as Hedysari polysaccharides [RHP] and microglial cell exosome carrier berberine and palmatine [Exos-Ber/Pal]). We found that these compounds can improve cognitive and motor dysfunction by inhibiting neuroinflammation while exerting neuronal protection, but their low bioavailability, mechanistic complexity, and lack of clinical translational evidence remain challenges. In the future, a combination of multi-omics techniques, rigorously designed clinical trials, and interdisciplinary strategies will be required to promote the precise application of herbal medicines in neuroinflammation-related diseases.

## 1 Introduction

Neurological disorders are the leading cause of disability and the second leading cause of death worldwide (1). Their disease burden continues to increase because of population growth and aging, indicating that their prevention and management are inadequate; this may primarily stem from a lack of a clear understanding of their etiology (2). Disturbances in common molecular pathways including oxidative stress, excitotoxicity, mitochondrial dysfunction, and autophagy have been implicated in the progression of neurodegenerative disorders (3). Neuroinflammatory cascade responses have been identified as a common causative factor in various neurological diseases, including stroke, Alzheimer’s disease (AD), Parkinson’s disease (PD), and ischemic/traumatic brain injury (TBI), and are common thread linked to pathology (4, 5).

Neuroinflammation is a normal immune response within the central nervous system (CNS) to noxious stimuli such as infection, injury, or toxins, but can also be autoimmune. It is a major pathophysiological feature and a key cause of many CNS disorders (6, 7). Resident neuroglial cells, including microglia (the resident immune cells of the CNS), astrocytes, oligodendrocytes, and neurons, are involved in this process (6, 8–10). Multiple sclerosis (MS) is an autoimmune CNS disease characterized by persistent inflammation and demyelination (11, 12). In the early stages of MS, persistent microglial activation results in the production of proinflammatory cytokines. These in turn induce further microglial activation, exacerbating MS symptoms (13, 14). AD is another CNS disease closely associated with neuroinflammation; it is also closely related to various pathological factors including Aβ plaques, phosphorylated tau, proinflammatory cytokines, and oxidative stress, which can activate microglia and induce neuroinflammation (15, 16). Neuroinflammation is also an important pathological feature of PD, which is primarily characterized by CNS microglial activation and proinflammatory mediator release; this inflammatory cascade results in progressive loss of dopaminergic neurons and exacerbates motor dysfunction (17, 18). Stroke is a severe CNS disease characterized by high morbidity and mortality rates. Microglia are activated, undergo morphological changes, and secrete cytokines within minutes of a stroke (19); in addition, astrocytes promote neuroinflammation by recruiting peripheral immune cells and releasing proinflammatory cytokines and chemokines (20, 21). Neuroinflammation is increasingly prevalent in patients with neurological disorders, and targeting it to modulate neurological disorders has important clinical applications.

Herbal medicines have a long history of treating various diseases and have been widely used as adjunctive therapies in clinical settings in Asian countries such as China, Japan, and Korea (22). However, their ambiguous pharmacological mechanisms have limited their development (23). Their advantages, such as multi-target mechanisms of action and favorable safety profiles, have brought these compounds into the limelight. Their pharmacological effects have been investigated by examining their active components (24, 25). Bioactive compounds of plant origin are commonly used to treat neurological disorders owing to their anti-inflammatory, antioxidative, and anti-apoptotic activities (26, 27). Numerous clinical and experimental studies have validated the therapeutic effects of natural phytochemicals on neurological disorders through the inhibition of neuroinflammation (28). Berberine mitigates neuronal damage induced by Aβ in AD, and ginsenoside Rg1 improves blood–brain barrier (BBB) disruption and TBI (29). This paper summarizes the research progress on bioactive compounds of herbal origin to treat neurological diseases by inhibiting neuroinflammation, discussing how to improve their utilization and target them to specific mechanisms to provide therapeutic strategies and drug candidates.

## 2 Effect of herbs on inflammation-related signaling molecules

### 2.1 TLR4/MyD88/NF-κB pathway

Although several therapeutic techniques are currently available for controlling neurodegenerative disorders, these drugs are associated with a wide range of long-term side effects when used over time. The development of safe, multi-targeted, and effective drugs for the treatment of neurodegenerative diseases, particularly those derived from natural products, is of particular importance. Studies on neurodegenerative diseases have highlighted the critical role of NF-κB in neurons and microglia (30). When the NF-κB pathway is activated in microglia, it exerts secondary neurotoxicity by stimulating the secretion of reactive oxygen species (ROS) and pro-inflammatory cytokines, including TNF-*α*, IL-1β, and interferon-*γ* (31). The Toll-like receptor 4 (TLR4)/MyD88/NF-κB pathway is the central regulatory network involved in neuroinflammation. The MyD88/NF-κB pathway recognizes pathogen- and damage-associated molecular patterns, activating a downstream pro-inflammatory cascade (32).

Baicalein (5,6,7-trihydroxyketone; C15H10O5) is an important flavonoid primarily isolated from the roots of *Scutellaria baicalensis* Georgi (Labiatae). Previous studies have demonstrated that it possesses various pharmacological properties, including antioxidant, anti-inflammatory, and neuroprotective effects (33). Zhang et al. reported the novel role of baicalein in anti-neuroinflammation by inhibiting the production of proinflammatory cytokines, suppressing the activation of astrocytes and microglial cells, and blocking NF-κB and MAPK signaling. Additionally, in a microglia model of lipopolysaccharide (LPS) activation, baicalein reduced inflammatory mediators by inhibiting IκBα phosphorylation and p65 translocation, and down-regulated TLR4, which functions upstream of NF-κB signaling. Baicalein treatment prevented rotenone-induced brain damage through its anti-inflammatory effects (34).

Additionally, tretinoin lactone, a diterpenoid tricyclic oxide isolated from *Tripterygium wilfordii* Hook F (TWHF), demonstrates pharmacological activity against inflammatory, neurodegenerative, and neuropathic pain (35). Premkumar et al. were the first to observe that tretinoin inhibits poly (I:C) (a TLR3 agonist)-induced COX-2 and iNOS expression in mouse macrophages; this suggests that tretinoin may prevent inflammation by inhibiting the TLR3 pathway in macrophages (36).

Zhang et al. reported that *Panax ginseng* saponin R1 (NG-R1) protects against ischemic stroke (IS) through multiple pathways; it reduces intestinal permeability and inflammation by inhibiting the TLR4/MyD88/NF-κB signaling pathway and simultaneously affects the microbiota-gut-brain axis by reducing the abundance of pathogenic bacteria and restoring the levels of beneficial bacteria. Additionally, NG-R1 also leads to the restoration of tight junction protein expression in the brain, ensuring BBB integrity (37).

Salvianolic acids (SAs) are hydrophilic phenolic compounds derived from *Salvia miltiorrhiza*. SA for injection (SAFI) is a lyophilized powder intended for intravenous administration. Zhao et al. reported that SAs inhibit the NF-κB and MAPK pathways by suppressing TLR4/MyD88 and TNF-*α* signaling, reducing inflammatory factor production; they also modulate the polarization of astrocytes and microglia to attenuate neuroinflammation (38). Wang et al. reported a higher likelihood of good functional outcomes at 3 months in patients receiving intravenous Recombinant tissue-type plasminogen activator(rt-PA) combined with SAFI than in those receiving intravenous rt-PA alone. Additionally, the use of SAFI for 2 weeks has been associated with improved neurological recovery (39).

MyD88 serves as an intracellular adapter protein for nearly all TLRs. TLR3 functions as an adapter protein that uses TRIF as a signal transducer (39, 40); IL-1β has also been shown to be downstream of the proinflammatory effects of TLR3 in certain diseases (41). Zhang et al. reported that intrathecal injection of triptolide exerts an anti-inflammatory effect by inhibiting the TLR3/TRIF/IL-1β pathway, which may be a potential mechanism by which tretinoin attenuates neuropathic pain induced by peripheral nerve injury (42). Additionally, triptolide downregulated inflammatory mediators (NF-κB, Cox-2, NLRP3, IL-1β, and TNF-*α*) in LPS-treated (100 ng mL^−1^) C2C12 myotubes, suggesting that it prevents LPS-induced inflammation and skeletal muscle atrophy (43).

The aforementioned herbal components exert multi-targeted anti-neuroinflammatory effects by targeting the TLR/NF-κB pathway, regulating glial cell polarization, and repairing the gut-brain axis. However, their bioavailability and clinical translational efficiency require technical optimization.

### 2.2 NLRP3 inflammatory vesicles

The nucleotide-binding domain of the leucine-rich repeat-containing receptor family pyrin domain-containing 3 (NLRP3) inflammasome, which contains a pyrin structural domain, is the most extensively studied inflammasome; it is implicated in numerous autoimmune and inflammatory diseases (44). The NLRP3 inflammasome is a protein complex consisting of NLRP3, a cysteine aspartate-specific protease 1 precursor (pro-caspase-1), and apoptosis-associated speckled protein. The assembly of NLRP3 inflammatory vesicles results in the maturation of pro-caspase-1 into caspase-1, which subsequently activates scorch death execution protein gasdermin D (GSDMD), creating pores in the cell membrane that exacerbate the release of IL-1β and IL-18 to trigger a more severe inflammatory response (45, 46). The transcriptional silent information regulator 1 (SIRT1) and downstream peroxisome proliferator-activated receptor-*α* coactivator (PGC-1α) can inhibit neuroinflammation by suppressing the NLRP3 inflammasome activation (47–49). Responses mediated by SIRT1 are involved in a variety of physiological processes, including oxidative stress, inflammation, and apoptosis (50).

Rhodopsin is derived from various natural sources, including rhubarb (51); it exhibits a range of pharmacological effects, including anti-inflammatory (52), anticancer, and immunosuppressive (53) effects such as autophagy, apoptosis, and pyroptosis (54, 55). An *in vitro* study reported that rhodopsin decreases microglial activation (56). Cui et al. reported that rhodopsin may reduce inflammation and demyelination in an experimental autoimmune encephalomyelitis (EAE) rat model, likely through the SIRT1/PGC-1α/NLRP3 signaling pathway, whereas microglia in an EAE rat model exhibited attenuated inflammation and demyelination (57). Jiang et al. reported that rhodopsin inhibits the LPS/ATP-induced activation of NLRP3 inflammatory vesicles, blocks the cleavage of GSDMD, and suppresses LPS/ATP-induced cellular scorch death in BV2 cells; additionally, it decreased the levels of inflammatory mediators TNF-*α*, IL-18, and IL-1β, reduced HT-22 hippocampal neuronal apoptosis, and restored cell viability (58).

Curcumin is derived from turmeric and exhibits numerous pharmacological and biological activities, including anti-inflammatory properties (59). Xu et al. reported that curcumin prevented rotenone-induced PD by inhibiting the activation of microglial NLRP3 inflammasomes and attenuating mitochondrial dysfunction in mice (59). Cai et al. reported that curcumin affected histone deacetylase (HDAC) 6, which directly modulated NLRP3 acetylation and inhibited neuroinflammation, alleviating neuronal degeneration in a PD model (60).

Yang et al. reported that astragaloside IV significantly inhibited NFκB-mediated inflammatory vesicle activation of NLRP3 in MPTP mice *in vivo* and BV2 microglial cells; it also activates Nrf2, which negatively influences NLRP3 activation by inhibiting ROS-induced activation. These findings suggest that astragaloside IV protects dopaminergic neurons by inducing neuroinflammation and oxidative stress (61).

Li et al. reported that tensin alleviates neuroinflammation by suppressing the ADRA1/NF-κB/NLRP3 pathway. Additionally, it ameliorated the pathological state of tau proteins and restored neuronal and BBB structures and functions, enhancing learning and memory in 3xTg-AD mice (62). Zhao et al. reported that the combination of ginseng and *Ginkgo biloba* extract modulated NLRP3 inflammatory vesicles and the CAMK4/CREB pathway to ameliorate neuroinflammation and excitotoxicity in IS (63). Tongxinluo, a novel neuroprotective formula with anti-inflammatory properties, is recognized for its ability to stabilize vulnerable plaques in animal models and patients with myocardial infarction (64). Wang et al. reported that it also significantly alleviated astrocyte death following cerebral ischemia/reperfusion by down-regulating the expression of cleaved caspase-11/1, GSDMD, NLRP3, IL-6, and cleaved IL-1β (65). Subsequently, a randomized clinical trial by Dong et al. demonstrated that in patients with IS within 72 h of symptom onset, those who received additional concentric loops were more likely to have a good functional outcome than the placebo group. These findings provide novel and valuable insights for the development of targeted therapeutic strategies for neurological disorders.

### 2.3 JAK–STAT signaling regulation

The overactivation of microglia and astrocytes exacerbates the involvement of the Janus kinase (JAK)/STAT pathway in neuroinflammatory diseases by initiating innate immunity, orchestrating adaptive immune responses, and suppressing inflammation and immune activity. The JAK/STAT signaling pathway is a pivotal driver of neuroinflammation in neurodegenerative disorders. Targeting this pathway through interventions such as JAK inhibitors holds significant therapeutic promise for treating conditions such as AD and MS (66). In a landmark discovery, Su et al. demonstrated that JAK1/STAT3 signaling serves as a pivotal regulator of neuronal cell proliferation, differentiation, and programmed cell death, while also exerting profound effects on inflammatory response mechanisms (67).

Echinacoside (ECH) is a phenylacetaldehyde glycoside isolated from the extract of *Dioscorea alata*; it has been extensively studied and found to have many pharmacological effects, such as antioxidant, anti-inflammatory, anti-infective, and anti-tumor effects (68, 69). Lu et al. demonstrated that both ECH and pinealoside significantly increased the ratios of p-JAK1/JAK1 and p-STAT3/STAT3. This compelling evidence indicates that pinealoside directly activates the JAK1/STAT3 signaling cascade, stimulating neuronal proliferation while concurrently inhibiting neuroinflammatory responses, ultimately manifesting potent antidepressant effects (70). Nakamura et al. revealed that sustained STAT3 signaling in senescent macrophages orchestrates microglial M2 polarization and significantly promotes neovascularization (71).

*G. biloba* (Ginkgoaceae), a reverse therapeutic agent for inflammatory bowel disease, is a potent herbal medicine used to treat IS. Its efficacy stems from key bioactive compounds such as flavonoid glycoside ligands and terpene lactones. Extensive pharmacological research has revealed that the active constituents of *G. biloba* exert neuroprotective effects in IS by combating inflammation, counteracting oxidative stress, and inhibiting apoptotic pathways while simultaneously stimulating neurovascular regeneration and enhancing axonal remodeling (72). Zhang et al. revealed in a groundbreaking study that *G. biloba* extract combats ischemic brain damage through dual mechanisms at the molecular level; it suppresses astrocyte proliferation and leverages the LCN2-JAK2/STAT3 pathway to inhibit neuroinflammatory cascades (73).

Although STAT3 is primarily activated by non-receptor tyrosine kinases of the JAK family, the activity of JAK itself is subject to tight regulation by the signal transduction inhibitory factor (SOCS) family (74). Paeoniflorin (PF), a monoterpene glucoside with therapeutic potential, is one of the most prominent bioactive constituents derived from *Paeoniflora* roots; its potent anti-inflammatory properties have been extensively documented in numerous animal studies revealing its efficacy in mitigating inflammatory responses (75). In a groundbreaking study conducted by Shi et al., researchers found that PF significantly upregulates the expression of cytokine SOCS3, effectively suppressing the IL-6/STAT3 signaling pathway in dendritic cells (DCs) (76). Additionally, Zhang et al. revealed that PF decreases Th17 differentiation by suppressing STAT3 phosphorylation. Their research demonstrated that PF not only inhibited IL-6 production in DCs but also lowered clinical scores in EAE mice, simultaneously delaying disease progression while maintaining cellular regulatory precision (77).

*Salvia divinorum* is derived from the rhizome of a traditional Chinese medicinal herb of the same name, and salvinorin IIA is a prominent lipophilic bioactive component. Chen et al. demonstrated that tanshinone IIA (TAN) suppresses JAK2 kinase activity, effectively inhibiting STAT1 Ser727 phosphorylation, thereby modulating this critical signaling pathway (78). Herbal active ingredients, including pineoside, paeoniflorin, and tanshinone IIA, exhibit high efficacy in orchestrating a delicate balance between neuroinflammatory processes and immune responses through dual-directional modulation of the JAK–STAT pathway (selectively activating or inhibiting distinct isoforms). However, challenges persist in elucidating their multi-targeting mechanisms and optimizing effective drug delivery systems.

### 2.4 Epigenetic regulation

The primary mechanisms of epigenetic dynamics include DNA methylation, histone modification, and non-coding RNA (79). DNA methylation is a reversible, heritable epigenetic modification that provides an additional layer of control over gene expression without changing the DNA sequence (80). Epigallocatechin-3-gallate (EGCG) is an extract that is the primary polyphenolic component of green tea (81). Klotho is an antioxidant, antifibrotic, and anti-inflammatory protein whose promoter is susceptible to DNA methylation. Yang et al. reported that under high glucose conditions, EGCG reduces the methylation of the Klotho gene promoter through DNA methyltransferase 3a to up-regulate Klotho expression and lower IL-1β, IL-6, and TNF-*α* levels (82).

Sirtuin is a widely present NAD + -dependent histone deacetylase (83). Liu et al. reported that ginsenoside Rg3 inhibits the NF-kB pathway by activating SIRT1, alleviating neuroinflammation and post-TBI in hippocampal neurons. These observations were further supported by *in vitro* experiments, which showed that ginsenoside Rg3 could attenuate hippocampal neuronal damage by inhibiting LPS-induced microglial activation through modulation of the SIRT1/NF-kB pathway (84). Additionally, within the spinal cord, the administration of rhodopsin decreases leukocyte infiltration, down-regulates IL-1β, reduces HDAC6 activity, and attenuates the interactions of HDAC6 with NLRP3; this decreases the activity of the HDAC6-NLRP3 complex, suppressing NLRP3 inflammatory vesicle responses to reduce spinal inflammation and chronic inflammatory pain (85).

miRNAs are small non-coding RNAs consisting of 18–25 nucleotides that regulate gene expression by binding to the 3′-UTR region of mRNAs, resulting in either the inhibition of translation or the induction of mRNA degradation (86). Using the TargetScan online database, Ding et al. predicted that the target gene of miR-182-5p was Rac1. Previous studies have indicated that activated Rac1 subsequently activates NF-κB and NOX2, resulting in increased inflammation, oxidative stress, and neuronal death (87). Berberine is an isoquinoline-derived alkaloid obtained from herbs such as *Berberis vulgaris* and *Phellodendron amurense*, which have traditionally been used to treat intestinal infections (87). Numerous studies have indicated that berberine exhibits neuroprotective effects in CNS disorders such as IS (88), AD (89), and PD (90). Ding et al. reported that berberine can act on damaged Rac1 in neurons to attenuate neuroinflammation (87). In conclusion, epigenetic regulation plays a significant role in the anti-inflammatory effects of herbal components.

## 3 Herbs and inflammation-associated cells

### 3.1 Microglial phenotype switching

A prevailing consensus indicates that microglial-mediated neuroinflammation is linked to neurodegenerative diseases, including AD, PD, and MS, exhibiting common pathophysiological mechanisms (91). Microglia are classified into neurotoxic M1 and neuroprotective M2 phenotypes (91). Microglia of the M1 phenotype release proinflammatory mediators, including nitric oxide (92), IL-1β, and TNF-*α*, leading to neurotoxicity and myelin damage. In contrast, M2 phenotype microglia promote the release of neurotrophic molecules and anti-inflammatory cytokines, such as insulin-like growth factor-1, glial cell-derived neurotrophic factor, and brain-derived neurotrophic factor (93–95). These molecules promote the differentiation of oligodendrocyte progenitor cells and enhance neuroprotection and myelin repair.

Considering the distinct roles of M1 and M2 microglia and macrophages, functional phenotypic modulators have been used as potential therapeutic agents for neurodegenerative diseases (96). Astragaloside IV (AST-IV) is a monomeric compound found in *Astragalus membranaceus*. Recent studies have demonstrated its neuroprotective effects in various intermediate neurological disorders including ischemia, Parkinson’s disease, Alzheimer’s disease, and autoimmune encephalomyelitis. AST IV alleviates motor deficits and enhances neurochemical activity by reducing inflammation and oxidative stress (97). Yu et al. reported that AST IV ameliorated paralysis and pathology in EAE by inhibiting neurotoxicity caused by M1 microglia, facilitating the shift to the M2 phenotype, and protecting neurons from apoptosis through inhibition of TLR 4/Myd 88/NF-κB signaling (98). Chen et al. reported that tanshinone IIA shifts the polarization of microglia to the M2 state by activating ERβ/IL-10 signaling; additionally, it attenuates neuronal loss and neuroinflammatory responses in mice with TBI (98). Sodium tanshinone sulfonate IIA (STS), a derivative of tanshinone IIA, possesses anti-inflammatory and anti-nociceptive properties. MiR-125b-5p is an immune-related miRNA that is highly expressed in microglia (99). Zeng et al. reported that STS pretreatment inhibits LPS-stimulated proinflammatory cytokine secretion, decreases proteins associated with the STAT3 pathway and apoptosis, increases miR-125b-5p and proopiomelanocortin expression, and enhances the conversion of microglial cells in BV-2 cells from the M1 to the M2 phenotype. STS exerts antinociceptive and antineuroinflammatory effects on neuropathic pain in neuropathic pain rats by targeting multiple pathways (99).

*Rhodiola rosea* (SLDS) extract, a phenylpropane glycoside extracted from the plant’s roots, is one of the main active components of the plant. Liu et al. reported that treating M1 microglia with SLDS promotes oligodendrocyte differentiation by transitioning from the M1 to the M2 phenotype, indicating that it may facilitate myelin regeneration in neurological diseases (100). IG et al. reported that *Artemisia absinthium* extract ameliorated excessive neuroinflammation and Aβ accumulation by modulating microglial activation and the autophagy-lysosome pathway, suggesting that it is a promising therapeutic candidate for the treatment of AD (101). These findings offer new avenues for treating neurological disorders.

### 3.2 Regulation of astrocyte function

Similar to the activation of microglia, and in line with the functional significance of their beneficial or harmful effects, reactive astrocytes are classified as either neurotoxic (A1) or neurotrophic (A2). Yu et al. reported that AST IV ameliorates paralysis in EAE by shifting astrocytes towards the neuroprotective A2 phenotype, protecting neurons from apoptosis and pathology (96). Aspalathin (4-hydroxybenzyl alcohol-4-O-*β*-D-glucopyranoside), a phenolic glycoside derived from the rhizome of the plant *Aspalathus*, has demonstrated various effects in preclinical models of CNS disorders. These include antioxidant properties, anti-inflammatory effects, and microglial cell activation inhibition (102). Wang et al. reported that aspalathin inhibits the development of microglial cells and astrocytes, reduced oxidative stress, and prevented neuronal apoptosis, preventing early brain injury induced by subarachnoid hemorrhage (103). A recent study by Zuo et al. suggested that aspalathin modulates astrocyte phenotypic changes through angiotensin type II1, indicating that it exerts therapeutic effects by modulating the RAS-SIRT3 pathway (104). However, the specific modulation mechanism remains unclear and should be the focus of future research.

### 3.3 Herbs and the gut-brain axis

Current research suggests that the gut flora contributes to microglial maturation, BBB development, and neuron proliferation, which are critical for the gut-brain axis (105). In addition, neurological disorders such as MS, PD, and AD are associated with the gut microbiome (105–107). Sun et al. reported a significant increase in the population of lactic acid bacteria in the intestines of AD mice following intervention with berberine, reducing intestinal inflammation and helping to maintain the balance of the intestinal microbiota (106, 108). Additionally, they conducted an immunofluorescence chemical analysis of mouse intestinal tissues and observed that berberine significantly increased the expression of the intestinal junction proteins ZO-1, occludin, and claudin-1, improving intestinal permeability and preventing endotoxins from entering the bloodstream through the intestinal barrier. Agirman et al. have shown that intestinal disorders favor humoral signaling of inflammatory factors through the gut-brain axis and can alter intestinal permeability and cause neuroinflammatory symptoms before changes in the CNS immune system (109). Berberine intervention may produce neuroinflammatory symptoms by altering the intestinal flora and increasing intestinal permeability, reducing brain inflammation to exhibit neuroprotection. Berberine also removes Aβ plaques and increases the number of neurons in the brain, alleviating AD to some extent (105).

Hedysari polysaccharide (RHP) is a key bioactive component of Radix Hedysari. Studies have demonstrated that RHP has neuroprotective properties (110). Yang et al. have reported that it modulates the hippocampal proteomic and serum metabolomic profiles of AD mice, enhances the intestinal barrier, attenuates neuroinflammatory responses, and reduces neuronal mitochondrial damage. This suggests that RHP ameliorates cognitive impairment in Senescence-Accelerated Mouse Prone 8 by regulating the gut-brain axis (111). *Pseudostellaria heterophylla*, a herb with a history of use spanning thousands of years in China, has been shown through modern pharmacological studies to possess various biological activities, including anti-inflammatory and immunomodulatory effects (112–114). He et al. have reported that Pseudostellaria heterophylla polysaccharide is a potent and effective drug for treating neuroinflammatory diseases in SAMP8 mice. They suggested that PH-PS might prevent AD progression by modulating the gut microbiota and glial polarization, offering evidence that could inform the design of potential dietary therapies to prevent or cure AD (115).

**Figure 1:**
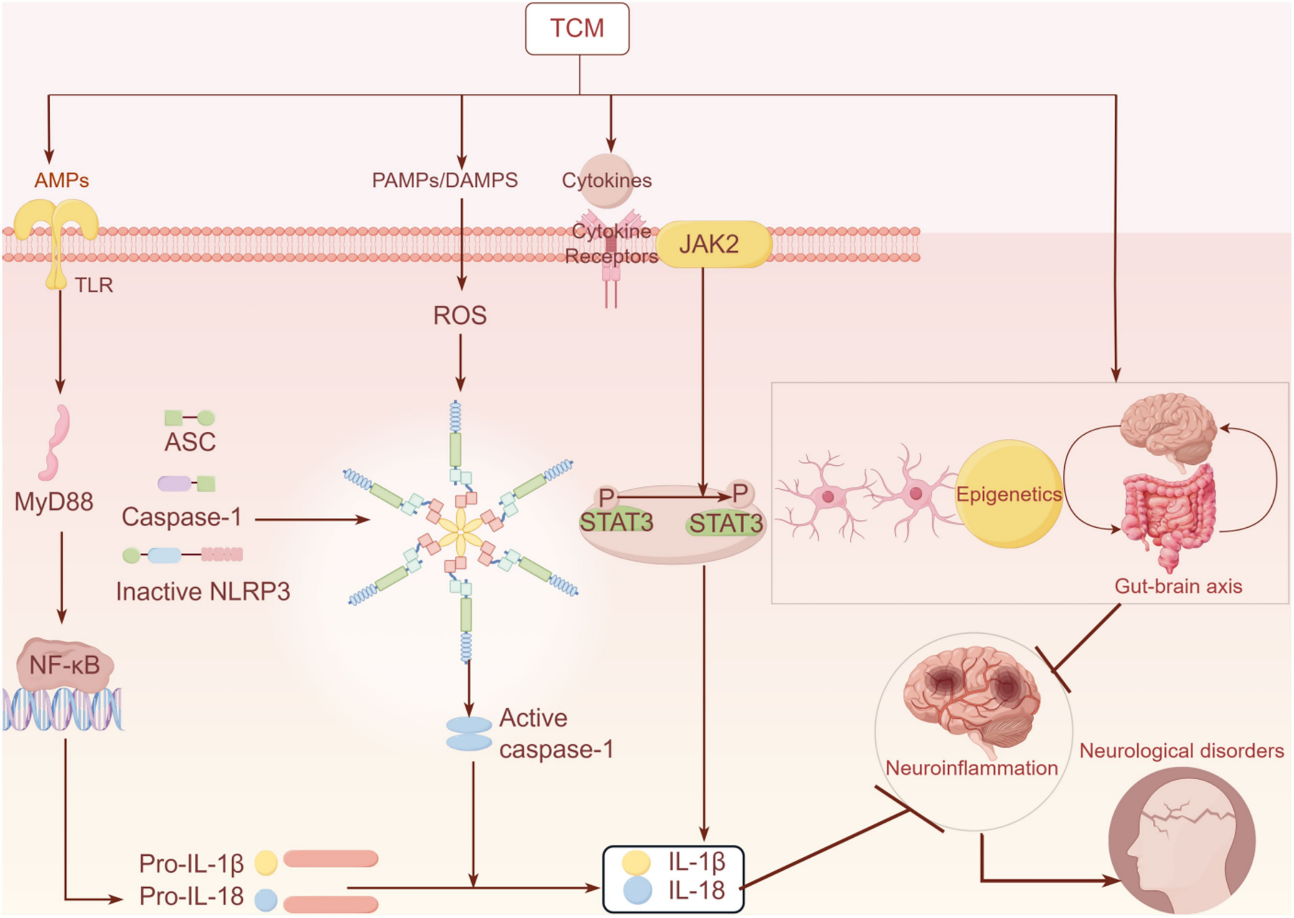
Related mechanisms of Chinese herbal medicine affecting inflammatory pathways.

Research has also focused on TCM prescriptions. Pingweisanjia Pharmaceutical (PWP) not only prevents the spread of *α*-synuclein across the gut-brain axis but also prevents neurodegeneration and behavioral deficits. PWP treats PD through multiple pathways, increasing beneficial flora involved in the gut-brain axis, including Actinobacteria and Lactobacillus as well as decreasing the expression of NLRP6 and GSDMD in PD mice (116–118). Despite the promising results shown, the understanding of how herbs affect the gut-brain axis is still unclear, providing a direction for future research (Figure 1).

Chinese herbal medicine can inhibit the release of inflammatory mediators, such as IL-1β and IL-18, by regulating related inflammatory pathways, (including TLR4/NF-κB, NLRP3, and JAK–STAT). Additionally, it can also suppress neuroinflammation by modulating the phenotypic transformation of astrocytes and microglia, as well as by regulating epigenetics and the gut-brain axis, thereby improving related neurological diseases.

## 4 Discussion

TCMs have demonstrated significant potential for treating neurological disorders owing to their multi-target and multi-mechanism actions. However, the bioavailability of most TCMs is poor after oral administration, necessitating further in-depth studies on targeted delivery (119, 120). Zhao et al. reported that a microglia-derived Exos-Ber/Pal delivery system enhances drug targeting and penetration into the brain. Additionally, the combination of berberine and palmatine was found to more effectively restore neurons, inhibit Aβ phagocytosis and microglial activation, and modulate the secretion of inflammatory factors (121). Hassan et al. reported the use of CS-TAN-NLCs (nanostructured lipid carriers) as an effective nano-agent for the treatment of PD following intranasal administration. The final results indicated that CS-TAN-NLCs improved exercise and alleviated depression in patients with PD, reducing NF-kβ and histone B expression to a greater extent than other delivery methods. Elevated levels of histone B can lead to the production of pro-inflammatory mediators and mitochondria-derived ROS, ultimately inducing neuronal death. Overall, CS-TAN-NLCs provide a highly adaptive strategy for the effective intranasal brain delivery of TAN for the treatment of PD (122). Yang et al. developed targeted liposomes and found that IGF1R-targeted salvianolic acid A -loaded liposomes demonstrated a more potent anti-neuroinflammatory effect than free SAA by suppressing the activation of microglia and the release of pro-inflammatory cytokines; additionally, it exhibited superior anti-neuroinflammatory effects and maintained good biosafety (123).

In conclusion, bioactive compounds associated with TCM can affect the progression and outcome of neurological diseases by regulating pathways related to neuroinflammation, epigenetics, and the gut-brain axis. Consequently, TCM has the potential to offer new therapeutic options for treating and curing neurological diseases.
